# Impact of cesarean section scar on decision regret and body image: a cross-sectional study**

**DOI:** 10.1590/1806-9282.20251717

**Published:** 2026-04-03

**Authors:** Hilal Ozbek, Edanur Ebru Yıldız

**Affiliations:** 1 Tokat Gaziosmanpasa University, Faculty of Health Sciences – Tokat, Türkiye.

**Keywords:** Cesarean section, Scar, Cicatrix, Body image, Decision making, Postpartum period

## Abstract

**OBJECTIVE:**

Physical changes and scarring after cesarean section may negatively affect women’s body image. Combined with decision regret about the mode of delivery, this may cause psychological problems. The aim of this study was to examine the effects of cesarean scars on decision regret and body image in women with cesarean delivery.

**METHODS:**

This descriptive and cross-sectional study was conducted in Turkey between February and May 2025. A total of 219 women with a history of cesarean delivery voluntarily participated through an online survey. Data were collected using a personal information form, the Decision Regret Scale, and the Body Image Scale. Statistical analyses were performed using Statistical Package for the Social Sciences 22.0 software, including descriptive statistics, t-test, analysis of variance, correlation, and regression analyses.

**RESULTS:**

The mean age of participants was 34.90±7.96 years, and the mean time since the first cesarean was 9.38±8.31 years. The mean Decision Regret Scale score was 22.46±20.12, and the mean Body Image Scale total score was 50.22±16.71. Decision regret and body image significantly differed according to perceptions of scar appearance and its impact on body perception, social life, and psychological well-being (p<0.05). Regression analysis indicated that negative perceptions of scar appearance were the strongest predictor of decision regret (B=15.42, p<0.001).

**CONCLUSION:**

Cesarean section scars may negatively affect women’s body image and increase decision regret regarding the mode of delivery. Healthcare professionals should be aware of these psychosocial consequences and provide appropriate counseling and support services for women after cesarean section.

## INTRODUCTION

Cesarean section is a life-saving intervention in situations where the health of the mother or fetus is at risk^1^. Although the World Health Organization has aimed to keep the ideal cesarean rate within the range of 10–15% since 1985^2^, in Turkey, this rate is well above the recommended limits. Between 2018 and 2023, the cesarean rate in Turkey was reported as 57.55%^3^. In fact, although cesarean section is an intervention that should be performed in situations where vaginal delivery poses a risk for the mother or fetus, these rates have increased due to women’s fear of labor pain, concerns about vaginal birth, uncertainties surrounding the process, and the perceived advantages of cesarean delivery^4^. However, it is also an important point to be considered that the scars that develop after cesarean section affect women both physically and psychologically^5^. Especially changes in cosmetic appearance, persistent pain, movement limitations due to adhesion or contracture, and problems such as itching may negatively affect women’s quality of life and psychological recovery process^5,6^. Consequently, women have to cope not only with physiological changes but also with psychological and social effects^5^. In the literature, there are also findings related to body image disorders and low self-esteem after a cesarean section^6,7^. In addition, it is stated that body image satisfaction of women who deliver vaginally is higher than that of women who deliver by cesarean section^7,8^. However, regret and low body image related to the cesarean scar may also lead to postpartum problems such as difficulties with breastfeeding duration, mother–infant bonding problems, and negative effects on sexual life^9,10^. In this context, understanding the effects of the scar formed on the female body after cesarean section on body image and decision regret regarding the mode of delivery is an important issue that should not be overlooked by healthcare professionals working in the field of women’s health. Realizing this situation may contribute to a more holistic assessment of the psychosocial needs experienced by women in the postpartum period and the development of appropriate supportive interventions. It may also contribute to providing more holistic and sensitive care to women, especially in terms of midwives and nurses. Furthermore, it is expected that the findings obtained will support social awareness aimed at reducing unnecessary cesarean sections. The aim of this study was to examine the effects of cesarean section scars that develop after cesarean section on women’s body image and decision regret regarding the mode of delivery.

### Research hypotheses/questions

1. The cesarean scar has an effect on women’s regret about their decisions.
2. The cesarean scar affects body image.
3. The cesarean scar affects women’s decision regret and body image.

## METHODS

### Study design and setting

This descriptive and cross-sectional study was conducted between March and May 2025 through an online survey with the participation of women who had undergone cesarean delivery.

### Population and sample

Women in Turkey who had undergone cesarean delivery were recruited via social media using a snowball sampling method.

### Sample size

Based on a correlation reported in the literature^11^ (r=0.34, α=0.05, power=0.80), the minimum required sample size was 66. In the present study, 219 women participated, yielding a post-hoc power >0.99.

### Inclusion and exclusion criteria

Eligible participants were 18–49 years old, had ≥1 cesarean, spoke Turkish, and consented. Those with incomplete forms or severe psychiatric disorders were excluded.

### Variables

Dependent: Decision Regret Scale and Body Image Scale (BIS) scores.

Independent: cesarean scar perceptions, sociodemographic, and obstetric factors.

### Data collection

#### Personal ınformation form

The Personal Information Form included 23 questions on women’s sociodemographic and obstetric characteristics, developed based on a literature review.

The Decision Regret Scale^12,13^ consists of five items scored on a five-point Likert scale. Total scores range from 0 to 100, with higher scores indicating greater regret. The Turkish version demonstrated good reliability (Cronbach’s α=0.84).

#### Body Image Scale

The BIS^14^ is a five-point Likert instrument measuring body image in young adults. It has four subscales—Body Change, Positive Perception, Negative Perception, and Evaluation Sensitivity—with Cronbach’s α values ranging from 0.70 to 0.79. Although originally validated in young adults, the items are not age-specific, and the scale has been applied in various adult samples in the literature. In the present study, the BIS demonstrated excellent reliability (Cronbach’s α=0.87) among women aged 20–49 years, supporting its suitability for this broader adult population.

#### Ethical considerations

The study was approved by a regional Ethics Committee (Decision No. 04-48, March 04, 2025). All participants provided informed consent electronically via Google Forms before participation.

#### Bias

Selection bias may have occurred due to snowball sampling and social desirability bias since data were self-reported. To minimize these risks, surveys were anonymous, participation was voluntary, and withdrawal was allowed.

### Data analysis

Data were analyzed in Statistical Package for the Social Sciences 22.0 at p<0.05. Normality was checked by skewness/kurtosis (-2 to +2). Descriptive statistics (frequency, percentage, mean, standard deviation, min–max) were calculated. Group differences were tested with t-tests and analysis of variance (ANOVA); correlations with Pearson analysis; and cesarean scar effects with hierarchical regression.

## RESULTS

### Sociodemographic characteristics

The mean age of participants was 34.90±7.96 years, and the mean time since the first cesarean was 9.38±8.31 years. Of the women, 52.5% had an associate degree or higher, 50.2% were employed, and 52.1% had undergone two or more cesarean sections. The recovery period after cesarean was reported as 0–6 months by 77.2%, and 50.2% had more than 6 years since their first cesarean. Regarding perceptions of the scar, 72.2% did not consider it a problem, 66.2% reported that it negatively affected body perception, and 82.2% stated that it did not affect their social life. In addition, 74.0% reported no regret about their cesarean decision, 47.9% were satisfied with their body, 53.9% perceived physical changes in body image, 68.9% reported no psychological difficulties, and 75.3% stated that they did not want to make changes regarding their body.

### Scale scores

The mean Decision Regret Scale (DRS) score was 22.46±20.12, and the mean BIS total score was 50.22±16.71. Subscale means were: Somatic Complaints 6.21±2.60, Evaluation Sensitivity 12.37±6.60, Positive Perception 15.31±6.01, Negative Perception 11.57±6.60, and Body Change 9.24±4.91.

### Comparison of scale scores

DRS and BIS scores differed significantly according to recovery time, feelings about scar appearance, effects on body perception and social life, regret about the cesarean decision, body satisfaction, perceived body image changes, psychological difficulties, and desire to change the body (p<0.05). Bonferroni post-hoc tests confirmed the group differences (Table 1).

**Table 1:** Findings on body image and psychological status after cesarean section (n=219).

Sociodemographic characteristics	DRS Mean±SD	BIS Mean±SD	Test (df)	P
Post-cesarean recovery period				
0–6 months	20.88±19.85	49.29±16.23	t(217)=-2.15	0.033
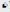 7 months	27.80±20.33	54.98±17.70	t(217)=-2.13	0.034
Feelings about the appearance of the cesarean scar				
Not a problem for me	16.55±16.81^a^	46.93±14.9^a^	F(2,216)=15.48	<0.001
A problem for me	37.56±20.58^b^	56.68±16.15^b^	F(2,216)=11.47	<0.001
Feel very bad about it	38.25±19.34^b^	60.90±20.38^b^		
Impact of cesarean scar appearance on body image				
Negatively affected	37.82±18.63	60.27±18.01	t(217)=8.94	<0.001
Affected	15.40±16.38	46.14±14.11	t(217)=6.31	<0.001
Impact of cesarean scar on social life				
Yes	40.51±18.77	63.00±17.86	t(217)=6.65	<0.001
No	18.55±18.22	47.90±15.21	t(217)=5.35	<0.001
Regret over the cesarean birth decision				
Yes	44.91±15.30	59.05±19.26	t(217)=4.65	<0.001
No	14.56±15.02	47.61±14.65	t(217)=2.06	0.040
Body satisfaction after cesarean				
Satisfied	15.74±16.79^a^	45.90±13.81^b^	F(2,216)=16.07	<0.001
Not satisfied	31.85±20.73^b^	57.90±19.95^a^	F(2,216)=11.93	<0.001
Other	24.20±20.40^b^	50.15±13.20^b^		
Perception of change in body image after cesarean				
Feeling less self-confident	33.88±22.63^a^	66.40±19.87^a^	F(3,215)=4.97	0.002
Physical changes	21.38±19.43^b^	48.76±16.46^b^	F(3,215)=12.38	<0.001
Psychologically affected	26.34±20.02^b^	53.26±15.35^b^		
No change experienced	16.56±29.02^b^	44.75±9.24^b^		
Psychological difficulties due to bodily changes				
Yes	35.29±19.96	60.36±19.71	t(217)=5.42	<0.001
No	16.68±17.39	46.19±13.01	t(217)=6.63	<0.001
Desire to make changes regarding the body after cesarean				
Yes	32.87±20.17	60.62±17.74	t(217)=5.40	<0.001
No	19.06±18.96	47.30±15.01	t(217)=4.57	<0.001

Note: DRS: Decision Regret Scale; BIS: Body Image Scale; SD: standard deviation. Values are presented as mean±SD. Independent samples t-test and one-way ANOVA were applied. Groups sharing the same superscript letter do not differ significantly (Bonferroni post-hoc test, p<0.05). Superscript letters “a” and “b” indicate statistically significant differences between groups; values sharing the same letter are not significantly different (p<0.05).

### Correlation analysis

There was a weak but significant positive correlation between DRS and BIS total score (r=0.182, p<0.01). At the subscale level, Evaluation Sensitivity (r=0.310, p<0.01) and Negative Perception of the Body (r=0.229, p<0.01) were positively correlated with DRS, whereas Positive Perception of the Body was negatively correlated (r=-0.321, p<0.01). The Body Change subscale was weakly positively correlated with DRS (r=0.182, p<0.01). Effect sizes ranged from small to medium (Table 2).

**Table 2:** Pearson correlations between Decision Regret Scale and Body Image Scale total and subscale scores (n=219).

		M±SD	1	2	3	4	5	6
1	Decision Regret Scale	22.46±20.13	0.182**	0.310**	0.229**	-0.321**	0.261**	0.182**
2	Body Image Scale	50.59±16.71		1	0.846**	0.822**	0.116	0.819**
3	Sensitivity to evaluation	12.37±6.60			1	0.660**	-0.153*	0.692
4	Negative body perception	11.57±6.60				1	-0.278	0.670
5	Positive body perception	15.31±6.01					1	-0.175
6	Desire to change body	9.24±4.91						1

Note: Values in the cells represent Pearson’s r coefficients (two-tailed). p<0.05, p<0.01. Higher BIS total scores indicate more negative body image. SD: standard deviation. The symbols indicate levels of statistical significance: *p<0.05 and **p<0.01.

### Hierarchical regression analysis

In Model 1, including age, number of cesareans, year of first cesarean, and wound healing duration, the predictors were not significant [R^2^=0.027, Adj. R^2^=0.013, F(3,215)=1.96, p=0.121]. Wound healing duration was weakly significant (B=-6.89, p=0.034) but lost its effect in later models (Table 3).

**Table 3:** Hierarchical linear regression predicting decision regret (n=219).

Variable	B	SE	β	t	P	95%CI [Min, Max]	R^2^	ΔR^2^	Adj. R^2^
Block 1 (control variables)							0.027	–	0.013
Age	0.05	0.16	0.02	0.33	0.746	[-0.26, 0.36]			
Number of cesarean sections	3.16	2.54	0.08	1.25	0.214	[-1.84, 8.16]			
Post-cesarean recovery period	0.52	2.95	0.01	0.18	0.860	[-5.29, 6.33]			
Block 2 (psychosocial variables)							0.306	0.279	0.279
Impact of scar appearance on body perception^a^	15.42	3.79	0.36	4.07	<0.001	[7.95, 22.88]			
Impact of scar on social life^a^	6.96	4.15	0.13	1.68	0.096	[-1.23, 15.15]			
Post-cesarean body satisfaction—Other^a^	3.23	3.22	0.06	1.00	0.318	[-3.12, 9.57]			
Post-cesarean body satisfaction—No^a^	6.25	3.36	0.15	1.86	0.064	[-0.36, 12.87]			
Psychological difficulties due to bodily changes	3.20	3.83	0.07	0.84	0.405	[-4.36, 10.76]			
Block 3 (main predictor)							0.310	0.004	0.280
Body Image Scale	-0.08	0.08	-0.07	-1.09	0.277	[-0.24, 0.07]			

Final model statistics: F(9, 209)=10.42, p<0.001; R^2^=0.310; Adj. R^2^=0.280; SEE=17.08; Durbin-Watson=1.90. Note: B: unstandardized coefficient; SE: standard error; β: standardized coefficient; p: significance level; R^2^: model R-squared; Adj. R^2^: adjusted R-squared; ΔR^2^: change in R-squared; CI: confidence interval. Dependent variable=Decision Regret Scale (DRS) total score.

^a^Reference categories: appearance of scar=“not bothered”; effect on body perception=“no”; effect on social/emotional life=“no”; body satisfaction=“satisfied”; body image change=“no change”. All dichotomous variables were coded as 0=reference and 1=comparison. Bold values indicate statistically significant results (p<0.05).

In Model 2, scar-related beliefs were added, and model fit improved significantly (R^2^=0.290, Adj. R^2^=0.279, F=16.89, p<0.001). Belief that the scar affected body perception was the strongest predictor of decision regret (B=15.42, β=0.36, p<0.001, 95%CI 8.0–22.8). Belief that the scar affected social life was also significant (B=8.99, β=0.17, p=0.027). Uncertainty about body satisfaction was marginally significant (B=6.40, p=0.052; Table 3).

In Model 3, adding BIS total score did not significantly improve the model (R^2^=0.310, ΔF=1.19, p=0.277). BIS total was not a significant predictor (B=-0.084, p=0.277).

Model assumptions were met, no multicollinearity was detected, and the Durbin-Watson coefficient (1.90) indicated no autocorrelation (Table 3).

## DISCUSSION

This study examined the relationship between body image and decision regret in women after cesarean delivery. The findings showed that although most women did not perceive the cesarean scar as a problem in their social lives, their body perception was negatively affected. As body image scores decreased, decision regret significantly increased, suggesting that body perception in the postpartum period is an important determinant of women’s emotional evaluation of birth experiences. These results emphasize that childbirth should be addressed not only physiologically but also psychosocially.

The findings are largely consistent with previous studies. Johnson reported that while most mothers did not view the cesarean scar negatively in terms of aesthetics, some expressed dissatisfaction, similar to our results^15^. Almeida et al. and Jamali et al. found that impaired body perception after cesarean delivery increased decision regret^5,16^. Chen and Chang noted that women experienced postpartum body dissatisfaction, with cesarean delivery playing an important role^17^. Jena and Ao emphasized depression, guilt, and regret after cesarean delivery^18^. Hadizadeh-Talasaz et al. reported that regret levels were higher two months after cesarean compared to vaginal birth^19^. On the other hand, some studies reported that women did not perceive the scar as a serious problem. In contrast, this study demonstrated that even when the scar was not considered a social problem, it still negatively affected body perception^20,21^. Cultural factors, postpartum care practices, and methodological differences in measurement tools may explain these variations.

This study makes a unique contribution by addressing body image and decision regret together. The results highlight that impaired body image is not merely an aesthetic concern but also a significant factor influencing the psychological consequences of delivery decisions. The use of standardized scales and an adequate sample size can be considered strengths of the study.

However, several limitations should be noted. Due to the cross-sectional design, causal relationships cannot be established. Self-reported data may also be affected by social desirability bias. The use of snowball sampling through an online survey may have influenced sample representativeness, as women without internet access, those less active on social media, or those uncomfortable sharing personal information online may have been underrepresented; therefore, some perceptions may have been either overestimated or underestimated. Another limitation concerns the age range of the BIS. Although the scale was initially developed for young adults, its psychometric properties in mid-adult women have been less frequently examined; therefore, the findings should be interpreted with this consideration in mind, despite the high internal reliability observed in our sample. The single-region sampling approach limits the generalizability to broader cultural settings. Furthermore, the exclusion of psychosocial variables—such as depression, anxiety, and social support—restricts the ability to conduct a more holistic assessment, as these factors are known to influence both body image and decision regret in postpartum women. These variables were not included to minimize participant burden in the online survey and to avoid excessive questionnaire length. Future research should incorporate these psychosocial dimensions to provide a more comprehensive understanding of the emotional and cognitive processes associated with cesarean scar perception and decision regret. Despite these limitations, the study’s methodological rigor and clinical relevance strengthen the value of the findings.

The results underline the need for postpartum care to address not only physical recovery but also body image and psychosocial well-being. Midwives should consider women’s experiences of body perception and decision regret when providing counseling. Such an approach may support women’s adaptation and reduce negative emotions associated with birth. At the health policy level, integrating psychosocial aspects into postpartum programs could reduce the long-term psychological burden after cesarean delivery.

Future research should examine longitudinal changes in body image and decision regret after cesarean delivery. Comparative studies of vaginal versus cesarean births and qualitative studies could provide deeper insights. Including variables such as psychological resilience, partner support, and mental health would broaden understanding. Cross-cultural studies may also clarify the influence of cultural norms on women’s perceptions of cesarean scars and related psychological outcomes.

## CONCLUSION

This study demonstrated that negative body image after cesarean delivery was significantly associated with decision regret. Although most women did not perceive the scar as a social problem, its negative effect on body perception highlights the importance of addressing psychosocial as well as physical recovery. Midwives and other health professionals should incorporate body image and emotional experiences into postpartum counseling and support. Further studies are recommended to evaluate changes in body image and decision regret over time and across different cultural contexts.

## Data Availability

The datasets generated and/or analyzed during the current study are available from the corresponding author upon reasonable request.
